# Identifying Cell-Type Specific Genes and Expression Rules Based on Single-Cell Transcriptomic Atlas Data

**DOI:** 10.3389/fbioe.2020.00350

**Published:** 2020-04-29

**Authors:** Fei Yuan, XiaoYong Pan, Tao Zeng, Yu-Hang Zhang, Lei Chen, Zijun Gan, Tao Huang, Yu-Dong Cai

**Affiliations:** ^1^School of Life Sciences, Shanghai University, Shanghai, China; ^2^Department of Science and Technology, Binzhou Medical University Hospital, Binzhou, China; ^3^Institute of Image Processing and Pattern Recognition, Shanghai Jiao Tong University, and Key Laboratory of System Control and Information Processing, Ministry of Education of China, Shanghai, China; ^4^Key Laboratory of Systems Biology, Institute of Biochemistry and Cell Biology, Chinese Academy of Sciences, Shanghai, China; ^5^Shanghai Institute of Nutrition and Health, Shanghai Institutes for Biological Sciences, Chinese Academy of Sciences, Shanghai, China; ^6^College of Information Engineering, Shanghai Maritime University, Shanghai, China; ^7^Shanghai Key Laboratory of Pure Mathematics and Mathematical Practice, East China Normal University, Shanghai, China

**Keywords:** cell type, expression rule, single-cell transcriptomics, tissue development, multi-class classification

## Abstract

Single-cell sequencing technologies have emerged to address new and longstanding biological and biomedical questions. Previous studies focused on the analysis of bulk tissue samples composed of millions of cells. However, the genomes within the cells of an individual multicellular organism are not always the same. In this study, we aimed to identify the crucial and characteristically expressed genes that may play functional roles in tissue development and organogenesis, by analyzing a single-cell transcriptomic atlas of mice. We identified the most relevant gene features and decision rules classifying 18 cell categories, providing a list of genes that may perform important functions in the process of tissue development because of their tissue-specific expression patterns. These genes may serve as biomarkers to identify the origin of unknown cell subgroups so as to recognize specific cell stages/states during the dynamic process, and also be applied as potential therapy targets for developmental disorders.

## Introduction

The increasing development of next-generation sequencing technologies has prompted great research progress in the areas of genomics, epigenomics, and transcriptomics (Schuster, 2007). Numerous notable achievements have been made through macro-scale studies. Nevertheless, scientists have begun to focus on the subtle differences among individual cells originating from the same organ or tissue to identify cellular heterogeneity, which plays crucial functional roles in cancers or other complex diseases (Meacham and Morrison, 2013). Cutting-edge single-cell sequencing technologies have emerged to address longstanding biological and biomedical questions.

The human body is composed of approximately 10^13^ single cells that live harmoniously in various sites and tissues (Bianconi et al., 2013). Each single cell is the fundamental unit of living organisms, and it plays a unique role in maintaining normal biological processes. In diseases such as cancer, the abnormal alteration of one single cell can initiate the progression of tumorigenesis and the subsequent downfall of the entire organism (Nowell, 1976). Previous studies usually focused on the analysis of bulk tissue samples, which are composed of millions of cells, to elucidate the mechanism and establish therapeutic strategies for treating diseases. However, the genomes within the cells of an individual multicellular organism are not always the same. Hence, identifying the key factors from averaged data sets is difficult. The recent developments in single cell sequencing techniques have provided insights into the detailed and comprehensive research of individual cells (Grün and van Oudenaarden, 2015).

Identifying cell components and cell types to understand cell functions is important because many organs comprise cells of various types and with interdependent functions. In addition, cell functions vary depending on the cells’ active or inhibited state, and they cause changes during organ development (Serewko et al., 2002). These factors cause huge challenges in classifying and cataloging the various cells in the human body. All adult diverse cells originate from a single zygote through a series of cell divisions and fate decisions in which one cell transitions from one type to another. The changes during embryonic development are driven by intricate gene expression programming (Maston et al., 2006), which reveals specific expression patterns in different types of cells at different development stages. At present, we can assay the expression profiles of every gene within genomes across thousands of individual cells in one experiment. Hence, we are capable of rigorously classifying cell types, defining the potential function of each cell type, and predicting the behavior of cells during biological development.

Many important genes play crucial roles in tissue development or cell differentiation with specific expression patterns. For instance, laminin can mediate tissue-specific gene expression in mammary epithelia in the presence of lactogenic hormones (Streuli et al., 1995). The expression level of transcription factor from zinc finger family turns out to be stable in hematopoietic stem cells but they turns out to have quite different expression patterns in the differentiated cells like erythroid cells, and megakaryocytes (Orkin, 2004). In various mesoderm- and endoderm-derived tissues, genes in the GATA family play a critical role in adjusting tissue-specific gene expression (Kelley et al., 1993; Laverriere et al., 1994). The expression levels of toll-like receptors and some related genes, such as CD14, MyD88, and LY96, vary across different adult human tissues, including the brain, heart, placenta, prostate, and trachea (Nishimura and Naito, 2005). These genes and their specific expression patterns during development and differentiation may be applied as biomarkers to recognize specific cell stages/states during the dynamic process.

On the basis of existing single-cell profiling datasets from a transcriptomic atlas of mice (Tabula Muris Consortium, 2018), we applied our newly presented computational approach to select crucial and characteristically expressed genes, which may perform essential functions in tissue development and organogenesis. We constructed some accurate classifiers that can group millions of cells into 18 tissue types depending on their gene expression profiles. We applied the minimum redundancy maximum relevance (mRMR) (Peng et al., 2005) and Monto Carlo feature selection (MCFS) (Draminski et al., 2008) methods to identify the most relevant gene features and decision rules classifying 18 cell categories and then ranked the features characterizing gene expression levels (Peng et al., 2005; Draminski et al., 2008). The selected features provided a meaningful list of genes that may have important functions during tissue development because of their specific expression patterns in distinct tissues. Further research of these genes may clarify the detailed mechanism of tissue development. In addition, these genes can be used as biomarkers to identify the origin of some unknown subgroups of cells. They can also be applied as potential targets for developmental disorders.

## Materials and Methods

### Datasets

We downloaded the single-cell expression profiles of 53,760 mouse cells in 18 tissues from Gene Expression Omnibus under accession number GSE109774 (Tabula Muris Consortium, 2018). The sample sizes of the tissues are listed in Table 1. The expression levels of 23,433 genes were measured using NovaSeq. We aimed to investigate the tissue differences at the single-cell level.

**TABLE 1:** Sample size of each tissue.

Index	Tissue	Sample size
1	Bladder	1638
2	Brain microglia	4762
3	Brain neurons	5799
4	Colon	4149
5	Fat	5862
6	Heart	7115
7	Kidney	865
8	Liver	981
9	Lung	1923
10	Mammary	2663
11	Marrow	5355
12	Muscle	2102
13	Pancreas	1961
14	Skin	2464
15	Spleen	1718
16	Thymus	1580
17	Tongue	1432
18	Trachea	1391

### Feature Selection

We designed a rigorous feature selection procedure for evaluating features. The purpose was to remove unimportant features for classifying cells from different tissues and rank remaining features according to their importance. First, each cell was represented in a vector of expression values of 23,433 genes, which were reduced to 5,451 by discarding features with low mutual information (MI) to targets. Second, remaining features were further reduced to 3,384 by using Boruta feature selection (BFS) (Kursa and Rudnicki, 2010). Third, these features were ranked by using mRMR (Peng et al., 2005) and MCFS (Draminski et al., 2008), resulting in two feature lists, respectively. Finally, on the basis of the ranked feature lists, incremental feature selection (IFS) (Liu and Setiono, 1998) with a supervised classifier was used to select the optimum features for classifying different cell types.

#### Evaluating Features by MI

Important criteria should be designed to determine important features according to meaningful correlations between variables and outputs. The direct way to measure the importance of features was to evaluate their correlations to targets. MI is a widely used and accepted measurement to assess features in this regard. The MI value for two variables *x* and *y* can be calculated by

(1) $$I\,\left(x,y\right)=\iint p\,\left(x,y\right)\,\log\frac{p\,\left(x,y\right)}{p\,\left(x\right)\,p\,\left(y\right)}\,d\,x\,d\,y$$

where *p*(*x*) and *p*(*y*) stand for marginal probabilistic density, and *p*(*x*, *y*) stands for joint probabilistic density. Here, for each feature, we calculated its MI value to targets (class labels) and selected those with MI values larger than 0.02. Remaining features would be poured into the following feature selection steps.

#### Boruta Feature Selection

In this step, features with MI values > 0.02 were analyzed by BFS (Kursa and Rudnicki, 2010). It is a wrapper feature selection method based on random forest (RF) (Breiman, 2001) that evaluates feature importance by comparing the features with randomized ones. BFS is different from most of the other wrapper feature selection algorithms that achieve minimal errors for a supervised classifier on a small subset of features, that is, BFS selects all features that may be either strongly or weakly relevant to outcome variables.

BFS mainly creates a shuffled version of original features and then uses an RF classifier to measure the importance score of the combined shuffled and original features. Only those features with importance scores higher than those of the randomized features are selected, and these significantly correlated features are considered relevant to the outcome variables. The difference between the RF and BFS importance scores lies in the introduction of the statistical significance of variable importance. A random permutation procedure is repeated to obtain statistically robust important features. BFS proceeds as follows by repeating multiple iterations:

1. Randomness is added to the given data set by shuffling original features.
2. The shuffled data set and original data set are combined.
3. An RF classifier is trained on the combined data set, and the importance of each feature is evaluated.
4. The Z-scores of the original and shuffled features are calculated. The Z-scores of individual features are calculated as the mean of the importance scores divided by the standard error. Each real feature is evaluated in terms of whether it has a higher Z-score than the maximum shuffled feature. If so, this feature is tagged as important; otherwise, it is unimportant.
5. Finally, the algorithm stops when one of the two following conditions is met: (1) all features are either tagged as “unimportant” or “important”; (2) a predefined number of iterations is reached.

In this study, we used the Python implementation of BFS from https://github.com/scikit-learn-contrib/boruta_py, along with the default parameters. Selected features were evaluated by mRMR and MCFS methods, respectively.

#### Minimum Redundancy Maximum Relevance

mRMR (Peng et al., 2005; Chen et al., 2017, 2018; Li et al., 2019) is a feature selection method based on MI. The merit of this method is that it considers both the relevance between input features and targets and the redundancy between features themselves. To indicate the importance of features, they are ranked in a feature list, named mRMR feature list. The list is generated by repeatedly selecting features from the feature pool until all features have been selected. In detail, for any feature in the feature pool, calculate its MI value to targets and its average MI value to already-selected features. Then, the difference of above-mentioned two values is computed. The feature with maximum difference is selected and appended to the list. In this study, the mRMR feature list was denoted by *F*_*m*_.

#### Monto Carlo Feature Selection

Different from mRMR method, MCFS (Draminski et al., 2008; Cai et al., 2018; Li et al., 2018; Chen et al., 2019) method evaluates the importance of features in a completely different way. This method is based on decision trees. First, it generates *m* bootstrap sets and *t* feature subsets from the original dataset. Then, one tree is grown for each combination of *m* bootstrap sets and *t* feature subsets. In total, *m* × *t* decision trees are grown. On the basis of these decision trees, we calculated the relative importance (RI) score for each input feature. The RI score is calculated in terms of how frequent a feature is involved in growing the decision trees, which can be computed by:

(2) $$R\,I_{f}=\sum_{\tau =1}^{m\,t}{\left(w\,A\,c\,c\right)}^{u}\,I\,G\,\left(n_{f}\,\left(\tau \right)\right)\,{\left(\frac{n\,o.i\,n\,\,n_{f}\,\left(\tau \right)}{n\,o.i\,n\,\,\tau }\right)}^{v}$$

where *f* stands for a feature, *wAcc* indicates the weighted accuracy of the decision tree *τ*, *IG*(*n*_*f*_(τ)) is the information gain of node *n*_*f*_(τ), *no*.*in n*_*f*_(τ) is the number of samples in *n*_*f*_(τ), (*no*.*in* τ) represents the number of samples in tree τ. *u* and *v* are weighted factors, which is set to 1. Clearly, features with high RI values are more important than others. Accordingly, features were ranked in another feature list with the decreasing order of their RI values. For convenience, this list was denoted as *F*_*M*_.

#### Incremental Feature Selection

Although, according to the results of mRMR and MCFS methods, we can obtain two feature lists, it is still difficult to access the optimum feature subspace for a given classifier. In view of this, IFS (Liu and Setiono, 1998) integrated with a supervised classifier was employed to select the optimum number of features for the classifier, thereby constructing the optimum classifier. On the basis of the feature list (*F*_*m*_ or *F*_*M*_), a series of feature subsets with step 5 is generated, that is, the first feature subset has the top 5 features, the second feature subset has the top 10 features, and so on. Then, for each feature subset, a supervised classifier (e.g., RF) is trained on the samples consisting of the features from this feature subset, and the classifier is evaluated using 10-fold cross-validation (Kohavi, 1995). The classifier with the best performance is selected and termed the optimum classifier, and the features used for this classifier are called the optimum features.

### Random Forest

RF (Breiman, 2001) is a supervised classifier comprising multiple decision trees, each of which is grown from a bootstrap set and a feature subset randomly selected from original features. RF has been widely used for many biological applications (Pan et al., 2010; Zhao et al., 2018; Zhao R. et al., 2019; Zhao X. et al., 2019; Zhang et al., 2019). One advantage of RF is that it does not require much effort in hyperparameter optimization; in general, only default parameters are necessary.

### PART Rule Learning

Contrary to black-box machine learning models, rule learning methods can learn rules about making a prediction from the data, and these rules are easy to understand. The most widely used rules is the if–then rule; IF one condition is met, THEN a prediction is generated. These simple rules can assist experts in analyzing learned knowledge so that it is aligned with established facts.

In comparison with another widely used rule learning method RIPPER, PART (Frank and Witten, 1998) learns a rule at a time without global optimization, and it is considerably simple. PART generates multiple partial decision trees and combines the rules from the decision trees using the separate-and-conquer technique. A pruned decision tree is built, and then a rule set is generated. Under this rule set, each rule walks along each path from the root to a leaf. The separate-and-conquer technique generates a rule at a time. Then, the instances aligned with this rule are removed from the training set until all instances are covered by the learned rules. PART repeatedly grows partial decision trees instead of a fully explored tree, and each partial tree is grown as follows: (1) dividing the samples into subsets; (2) expanding all subsets until each subset is expanded to a leaf in the same way as C4.5, with the only difference being the selection of the node with the lowest entropy for expansion; and (3) backtracking is intrigued when all child nodes of internal nodes are expanded into a leaf. PART prunes the trees by checking if an internal node can be replaced with a leaf. Once a tree is built, a rule can be extracted from its leaf to the root.

## Results

**FIGURE 1:**
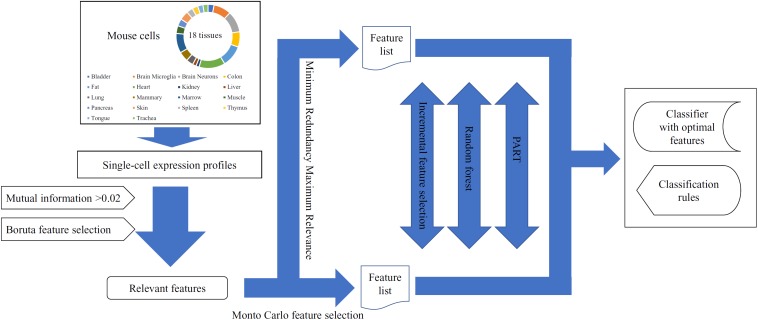
The entire procedures for analyzing the single-cell expression profiles of mouse cells in 18 tissues.

In this study, we used several machine learning algorithms to analyze the single-cell expression profiles of mouse cells in 18 tissues. The whole procedures are illustrated in Figure 1.

### Results of Feature Selection Procedure

There were more than 50,000 features to encode each mouse cell in 18 tissues. A rigorous feature selection procedure was necessary to analyze them. First, we evaluated the importance of each feature by its MI value to targets. Those with MI values larger than 0.02 were picked up, resulting in 5,451 features. Then, the BFS method was applied on the remaining features to further select relevant features, producing 3,384 features.

Above-obtained features were fed into mRMR and MCFS methods, respectively. Accordingly, we obtained two feature lists, which are summarized in Supplementary Tables S1, S2, respectively.

### Results of IFS With RF

The mRMR and MCFS methods provided different rankings of the remaining 3,384 features. We used IFS with RF to analyze the ranked features and thereby obtain the optimum features for classifying different cells with RF.

**FIGURE 2:**
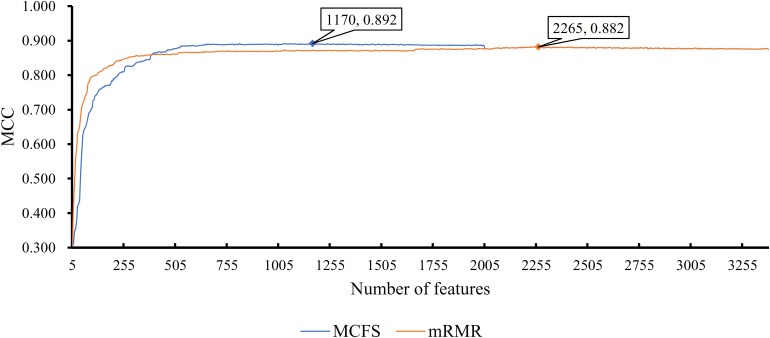
IFS curves for IFS with RF on the feature list yielded by mRMR and MCFS methods, respectively. The best MCC for RF on the list yielded by mRMR method is 0.882 when top 2265 features are used. The highest MCC for RF on the list yielded by MCFS method is 0.892 when top 1170 features are adopted.

**FIGURE 3:**
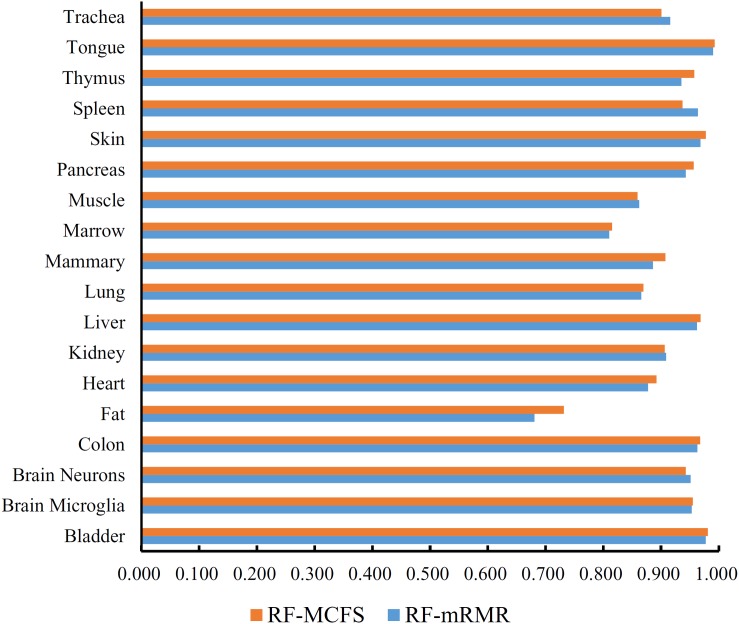
Bar chart to show accuracies on 18 tissues yielded by the optimum RF classifiers on the feature lists of mRMR and MCFS methods.

First, we applied IFS with RF to select the optimum features on the basis of the mRMR feature list yielded by mRMR method. Step five was adopted to construct a series of feature subsets. On each feature subset, one RF classifier was trained and evaluated on the samples consisting of the features from this feature subset by using 10-fold cross-validation (Kohavi, 1995; Che et al., 2019; Cui and Chen, 2019; Zhou et al., 2019). The performance corresponding to the different numbers of features is given in Supplementary Table S3. For an easy observation, an IFS curve was plotted in Figure 2 with Matthew’s correlation coefficient (MCC) (Matthews, 1975) as Y-axis and number of features as X-axis. We can see that when the top 2,265 features were used, the RF classifier yielded a maximum MCC value of 0.882 and an overall accuracy of 0.890 (Table 2). The performance of such optimum classifier on 18 tissues is shown in Figure 3. 12 tissues received accuracies over 0.900, suggesting the good performance of such classifier.

**TABLE 2:** Performance and optimum number of features of IFS with RF when using different feature ranking methods.

Feature ranking	Number of optimum features	MCC	Overall accuracy
mRMR	2265	0.882	0.890
MCFS	1170	0.892	0.899

We also applied IFS with RF to select the optimum features from the feature list produced by MCFS. The performance corresponding to the different numbers of features is provided in Supplementary Table S4. An IFS curve was also plotted in Figure 2 for clearly displaying the performance of RF classifier on different numbers of top features. When top 1,170 features were adopted, the RF classifier generated the highest MCC of 0.892 and overall accuracy of 0.899 (Table 2), which were a litter better than those of the optimum RF classifier on the feature list yielded by mRMR method. The detailed performance of such classifier on 18 tissues is illustrated in Figure 3. 13 tissues were assigned accuracies exceeding 0.900. These results indicate that this optimum RF classifier yielded better performance when using much fewer features from MCFS than from mRMR.

**FIGURE 4:**
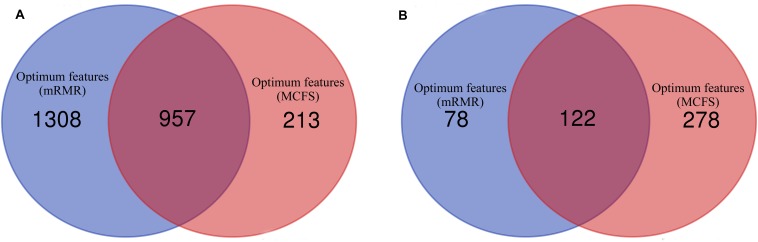
Venn diagrams to show the intersection of optimum features for RF and PART based on the feature lists of mRMR and MCFS methods. **(A)** Venn diagram to show the intersection of optimum features for RF; **(B)** Venn diagram to show the intersection of optimum features for PART.

As analyzed above, the optimum features for RF on the list yielded by mRMR method were top 2,265 features, and they were top 1,170 features for RF on the list yielded by MCFS method. A Venn diagram was plotted in Figure 4A to show the intersection of two optimum feature sets. There were 957 common feature (genes). We used hypergeometric test to assess their overlapping significance, obtaining *P*-value less than 0.05. Thus, these two feature select methods tend to output the same important features.

### Results of IFS With PART

**FIGURE 5:**
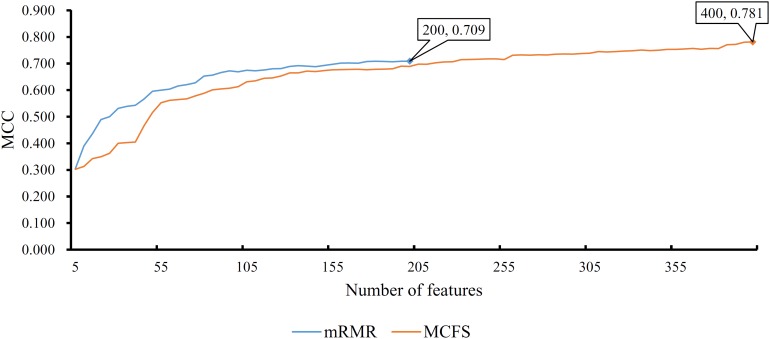
IFS curves for IFS with PART on the feature list yielded by mRMR and MCFS methods, respectively. The best MCC for PART on the list yielded by mRMR method is 0.709 when top 200 features are used. The highest MCC for PART on the list yielded by MCFS method is 0.781 when top 400 features are adopted.

**FIGURE 6:**
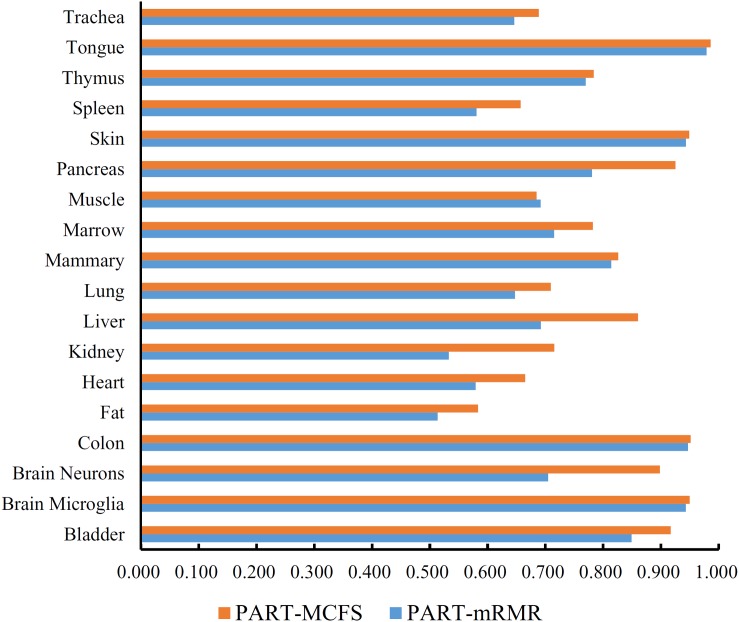
Bar chart to show accuracies on 18 tissues yielded by the optimum PART classifiers on the feature lists of mRMR and MCFS methods.

In addition to the use of the black-box classifier RF as the supervised classifier, the rule learning classifier PART is also utilized to select the optimum features for classifying different cells. Because PART is a rule learning algorithm with low efficiency, we only tried the top 200 features on the list of mRMR method. The 10-fold cross-validation results of PART classifier on different numbers of top features is listed in Supplementary Table S5. An IFS curve was plotted in Figure 5, from which we can see that the highest MCC was 0.709 when top 200 features were used. The overall accuracy was 0.730 (Table 3) and the detailed performance on 18 tissues is displayed in Figure 6. There were four tissues receiving accuracies higher than 0.900. All these suggest that such classifier provided an acceptable performance. Thus, the PART used these 200 features to construct rules based on all mouse cells, resulting in 7085 classification rules. These rules are listed in Supplementary Table S6.

**TABLE 3:** Performance and optimum number of features of IFS with PART when using different feature ranking methods.

Feature ranking	Number of optimum features	Number of classification rules	MCC	Overall accuracy
mRMR	200	7085	0.709	0.730
MCFS	400	7413	0.781	0.798

Similarly, we performed IFS with PART on the feature list from MCFS. We tried top 400 features this time. The performance of PART classifier corresponding to different numbers of top features is summarized in Supplementary Table S7. An IFS curve was plotted in Figure 5. It can be observed that when top 400 features were used, the PART classifier yielded the best MCC value of 0.781 and an overall accuracy of 0.798 (Table 3), which were higher than those of the PART classifier on the feature list of mRMR method. The detailed performance of such classifier on 18 tissues is shown in Figure 6. The accuracies on six tissues were higher than 0.900, also better than those of PART classifier generated by mRMR results. Furthermore, PART used obtained 400 features to build classification rules with all cells, generating 7,413 classification rules, which are listed in Supplementary Table S8.

Of the top 200 features in the mRMR feature list and top 400 features in the list of MCFS method, exactly 122 genes were common (Figure 4B). The overlapping significance on these two feature sets was at *P* < 0.05. Therefore, these two methods also tended to robustly select the same important features for PART.

## Discussion

In this study, the single-cell expression profiles of mouse cells in 18 tissues were analyzed by several machine learning algorithms. With two feature selection methods, mRMR and MCFS, two optimum RF classifiers were built and important genes were listed in two feature lists. However, the optimum RF classifiers were black-box classifiers, which can not reveal the different expression patterns of cells in different tissues. Thus, we further employed the rule learning algorithm, PART. With different feature selection methods, we obtained two groups of classification rules, which are provided in Supplementary Tables S6, S8. The first rule group (Supplementary Table S6) contained 7085 rules, involving 95 crucial features (genes) and the second group consisted of 7413 rules, using 130 crucial features (genes). In this section, we focused on some crucial features and decision rules with classification significance. These characteristics of gene expressions play key roles in tissue-specific differentiation or organ specificity.

### Analysis of Top Gene Features and Decision Rules Identified Using mRMR

We identified 7085 decision rules involving 95 features via the mRMR method to distinguish 18 different types of tissues. Here, we briefly summarized some experimental evidence for the most significant features and rules in the classifier to validate the efficacy and accuracy of our prediction.

The protein coding gene **Hexb**, which was identified as the most relevant feature through the mRMR method, produced the beta subunit of the lysosomal enzyme beta-hexosaminidase that can degrade various substrates containing N-acetylgalactosamine residues. Hexb transcripts distribute widespread tissues, thus playing a housekeeping role in the enzyme. However, the expression patterns of Hexb exhibit tissue-specific differences with relatively low levels in the lung, liver, and testis, which imply its unique biological function in tissue differentiation (Yamanaka et al., 1994). Similarly, another study analyzed the tissue distribution of the Hexb mRNA in mice and revealed remarkable tissue-specific variations, with the kidney showing the highest gene expression, which are consistent with past research (Triggs-Raine et al., 1994). These findings are consistent with our expectation that Hexb displays a restricted pattern in distinct tissues and is thus an effective feature in classification.

**Lgals7**, also known as Galectin7, is a member of beta-galactoside-binding proteins that are implicated in modulating cell–cell and cell–matrix interactions. Differential studies indicate that lectin is specifically expressed in keratinocytes and is mainly found in stratified squamous epithelium (Magnaldo et al., 1998; Saussez and Kiss, 2006). This finding confirms our decision rules that the high expression of Lgals7 leads to the identification of skin tissues. Meanwhile, the increased expression of Lgals7 plays a positive role in cell growth and dispersal by inducing MMP9 (Demers et al., 2005). However, the functional effects of Lgals7 vary across different tissue types, and thus, the multiple roles of Lgals7 may be tissue-type dependent (Shadeo et al., 2007).

Protein coding gene **Lgals4** or galection4, as another member of the beta-galactoside-binding protein family, has a similar function to galectin7 in protein interactions, but it shows a differential expression pattern that is restricted to the intestine, colon, and rectum (Huflejt et al., 1997). It is consistent with our decision rules, which require a high level of Lgals4 expression to classify cells into the category of the colon. Galectin4 is overexpressed mainly in cells with highly differentiated polarized monolayers but is absent in less differentiated ones, suggesting its crucial roles in organogenesis and its potential as a tissue-specific marker (Huflejt and Leffler, 2003).

The protein encoded by **Krt5** (keratin 5) is a member of the keratin gene family, which comprises cytoplasmic intermediate filament proteins that are usually expressed in epithelial tissues in a differentiation-dependent manner. Keratins display a complex expression pattern that is tightly regulated by the differentiation progress of the tissue in stratified epithelia (Alam et al., 2011). Gene ontology annotations related to Krt5 contain structural molecule activity, and mutations in this gene are associated with epidermolysis bullosa simplex (Schuilenga-Hut et al., 2003). KRT5 is one of the basal epithelial cell markers similar to KRT7 and EGFR, which follow several rules in our prediction in which Krt5 should have a low expression or even absent expression in fat tissue.

The purinergic receptor P2Y12 (**P2ry12**), which belongs to the family of P2 purinergic receptors, is a specific marker for microglial cells in the human brain (Sasaki et al., 2003). Microglial chemotaxis and the extension of microglial foot processes are significantly inhibited by P2ry12 deficiency and thus perform unique functions in microglia development (Haynes et al., 2006). Notably, a highly expressed pattern of P2ry12 contribute to the identification of brain microglia in our decision rules.

Another protein coding gene, **Ctsd** (Cathepsin D), produces a member of the A1 family of peptidases. Cathepsin is a marker of gastric differentiation, and its expression is significantly correlated with the originated histological type of gastric cancer cell line (Konno-Shimizu et al., 2013). This finding supports the potential role of Ctsd in gastric-related tissue specificity.

P53 apoptosis effector related to PMP22 (**Perp**) is a component of intercellular desmosome junctions. It plays a role in stratified epithelial integrity and cell–cell adhesion by promoting desmosome assembly (Ihrie et al., 2005; Kiseljak-Vassiliades et al., 2017). Perp plays an antiapoptotic role, and the loss of Perp function leads to strong apoptosis in the skin, indicating that this gene is required for the survival of specific cell types during development (Nowak et al., 2005). Notably, in the decision rules identifying heart tissues, several criteria that involve Perp, which require a relatively high expression of this gene, have experimental support. According to the immunohistochemical analysis, the Perp message is present in the intercalated discs of the cardiac muscle during embryogenesis but not in tissues containing simple epithelia, such as the lung. These results highlight the crucial role of Perp and the potential tissue-specific marker in stratified epithelia (Marques et al., 2006).

**Ptprcap**, also called Cd45-AP, is a transmembrane phosphoprotein that is associated with tyrosine phosphatase PRPRC/CD45, which can regulate T- and B-lymphocyte activation. It is overexpressed in PBMCs, which can enhance the phosphate activity of CD45 and increase tumor progression (Kitamura et al., 1995; Mao et al., 2008). It confirmed our predicted rules that the highly expressed pattern of Ptprcap is the indicator of marrow and thymus cell origin.

Legumain, also known as asparaginyl endopeptidase, which is encoded by the **Lgmn** gene, plays a role in the regulation of cell proliferation via its role in EGFR degradation and may be involved in the processing of proteins for MHC class II antigen presentation in the endosomal system (Manoury et al., 1998; Chen et al., 2001; Clerin et al., 2008). Legumain acts by regulating the differentiation fate of human bone marrow stromal cells, thereby regulating bone formation, which is independent of its enzymatic activity (Jafari et al., 2017). Legumain is overexpressed in bone marrow adipocytes, thereby supporting our decision rules regarding the classification of marrow, which require a highly expressed level of Lgmn, thus confirming the reliability of our predictor.

### Analysis of Top Gene Features and Decision Rules Identified Using MCFS

7413 decision rules, involving 130 crucial features, were identified by MCFS and PART methods. Among the top features with the most relevance in terms of classification, some features had biological evidence of their potential tissue-specific expression patterns, which can thus be applied as biomarkers for distinguishing cell origins.

Notably, many of the features mentioned previously, including **P2ry12**, **Krt5**, **Lgals7**, **Lgals4**, and **Hexb**, were identified by mRMR and MCFS methods and have a remarkable relevance to our classifiers. These results strongly suggest that these genes have significant tissue-specific patterns and exert an important effect on the classification of different tissue cells.

**DSC3** (Desmocollin 3), which ranks third among the relevant features identified by MCFS, may contribute to epidermal cell positioning by mediating the differential adhesiveness between cells that express different isoforms (Yue et al., 1995). In the decision rules for identifying lung and trachea tissues, Dsc3 should have a high expression level. RT-PCR results constantly showed that Dsc3 is expressed in the epithelium of the trachea and upregulated in the squamous cell in the lung (Nuber et al., 1996; Kettunen et al., 2004). Furthermore, desmosomal proteins are markers of epithelial differentiation (Moll et al., 1986). The expression pattern of Dsc3 changes with epidermal organization during skin development (Chidgey et al., 1997). Hence, Dsc3 may display specific expression patterns during cell differentiation and may thus support the process of distinguishing diverse stages of tissue development.

***Cdx1*** is a member of the caudal-related homeobox transcription factor gene family. The encoded DNA-binding protein regulates intestine-specific gene expression and enterocyte differentiation (Park et al., 2009). Homeobox genes are essential in the control of normal embryonic development. Recent publications on *Cdx1* suggested that early intestinal development, differentiation, and phenotype modulation are precisely regulated by effective transcription factors (Silberg et al., 2000). In addition, *Cdx1* is an important molecular mediator, which induces intestinal metaplasia in mouse stomach (Mutoh et al., 2004). These findings confirmed that in the criteria involving the decision rules for identifying colon tissues, highly expressed *Cdx1* indicates that the tissue may derived from colon associated tissues. In the same rules for identifying colon tissues, ***Gpx2***, which encodes the protein of the glutathione peroxidase family, requires a high expression like that of *Cdx1*. This gene is predominantly expressed in the gastrointestinal tract, and the overexpression of *Gpx2* is associated with increased differentiation and proliferation in colorectal cancer (Komatsu et al., 2001), thus contributing to colon development.

G protein-coupled receptors, such as **Gpr34**, mediate signals to the interior of the cell by activating heterotrimeric G proteins. Ubiquitous expression of Gpr34 is detectable in almost all human tissues; however the activity of promoters shows tissue-specific preference, which leads to different transcription patterns and various expression levels (Schöneberg et al., 1999). This special characteristic of Gpr34 allows its role in distinguishing different tissues and confirms that Gpr34 occurs in many decision rules with different criteria. Similarly, protein coding gene ***Cx3cr1***, which encodes fractalkine receptor, has diverse expression patterns in different cell types. The expression of *Cx3cr1* has been investigated in the mouse central nervous system, and its expression is elevated on microglia during chronic inflammation (Hughes et al., 2002). TGF-β1 plays an important role in regulating Cx3cr1 expression in rat microglia and inhibits fractalkine-stimulated signaling (Chen et al., 2002). The specific expression pattern of Cx3cr1 is consistent with our decision rules in which a high expression level indicates the category of brain microglia, although the criteria for identifying brain neurons require a low expression or absence of *Cx3cr1*.

Paired-like homeodomain 1 (**Pitx1**) encodes a member of the PITX homeobox family, which is involved in organ development and left-right asymmetry. This protein may act in the development of anterior structures and in specifying the identity or structure of hindlimbs (Logan and Tabin, 1999; Klopocki et al., 2012). Pitx1 exhibits the preferential expression in the hindlimb, and it critically modulates the potential patterning of specific hindlimb regions (Szeto et al., 1999). Pitx1 is expressed in lung epithelia cells, but its expression level varies during cancer development and progression, indicating that homeobox genes are associated with differentiation and show unique expression patterns at different development stages (Chen et al., 2007). It provides the basis for the use of Pitx1 as a potential biomarker.

Considering our single-cell profiling datasets, we carefully selected the crucial and characteristically expressed genes by using mRMR and MCFS, respectively, and their expression rules by using PART. These relevant gene features and decision rules may play essential roles in tissue development and organogenesis corresponding to 18 tissue types. Many biological studies about these may clarify the detailed mechanism of tissue development. Thus, our identified feature genes can be used as biomarkers to identify the origin of some unknown subgroups of cells, which can also be applied as potential therapy targets for developmental disorders.

## Conclusion

This study gave an investigation on single-cell expression profiles of mouse cells in 18 tissues using several machine learning algorithms. Some essential genes that can be biomarkers for distinguishing cells of different tissues were extracted by feature selection methods and two RF classifiers were built to classify cells with high performance. In addition, two rule groups yielded by PART were reported to reveal specific expression patterns of cells in different tissues. The findings reported in this study can give a clear overview on the expression levels of different tissues.

## Data Availability Statement

The datasets for this study can be found in the Gene Expression Omnibus (https://www.ncbi.nlm.nih.gov/geo/query/acc.cgi?acc=GSE109774).

## Author Contributions

TH and Y-DC designed the study. FY, XP, and LC performed the experiments. FY, TZ, Y-HZ, and ZG analyzed the results. FY and XP wrote the manuscript. All authors contributed to the research and reviewed the manuscript.

## Conflict of Interest

The authors declare that the research was conducted in the absence of any commercial or financial relationships that could be construed as a potential conflict of interest.
